# Intracellular localization of membrane-bound ATPases in the compartmentalized anammox bacterium ‘*Candidatus* Kuenenia stuttgartiensis’

**DOI:** 10.1111/j.1365-2958.2010.07242.x

**Published:** 2010-06-11

**Authors:** Laura van Niftrik, Mary van Helden, Silke Kirchen, Elly G van Donselaar, Harry R Harhangi, Richard I Webb, John A Fuerst, Huub J M Op den Camp, Mike S M Jetten, Marc Strous

**Affiliations:** 1 Department of Microbiology, Institute for Water and Wetland Research, Faculty of Science, Radboud University NijmegenHeyendaalseweg 135, 6525 AJ Nijmegen, the Netherlands; 2 Cellular Architecture & Dynamics, Utrecht UniversityPadualaan 8, 3584 CH Utrecht, the Netherlands; 3 Department of Microbiology & Parasitology (JAF)/Centre for Microscopy and Microanalysis (RIW), University of QueenslandBrisbane, Qld 4072, Australia; 4 Department of Biotechnology, Delft University of TechnologyJulianalaan 67, 2628 BC Delft, the Netherlands; 5 Max Planck Institute for Marine MicrobiologyCelciusstrasse 1, 28359 Bremen, Germany

## Abstract

Anaerobic ammonium-oxidizing (anammox) bacteria are divided into three compartments by bilayer membranes (from out- to inside): paryphoplasm, riboplasm and anammoxosome. It is proposed that the anammox reaction is performed by proteins located in the anammoxosome and on its membrane giving rise to a proton-motive-force and subsequent ATP synthesis by membrane-bound ATPases. To test this hypothesis, we investigated the location of membrane-bound ATPases in the anammox bacterium ‘*Candidatus* Kuenenia stuttgartiensis’. Four ATPase gene clusters were identified in the *K. stuttgartiensis* genome: one typical F-ATPase, two atypical F-ATPases and a prokaryotic V-ATPase. *K. stuttgartiensis* transcriptomic and proteomic analysis and immunoblotting using antisera directed at catalytic subunits of the ATPase gene clusters indicated that only the typical F-ATPase gene cluster most likely encoded a functional ATPase under these cultivation conditions. Immunogold localization showed that the typical F-ATPase was predominantly located on both the outermost and anammoxosome membrane and to a lesser extent on the middle membrane. This is consistent with the anammox physiology model, and confirms the status of the outermost cell membrane as cytoplasmic membrane. The occurrence of ATPase in the anammoxosome membrane suggests that anammox bacteria have evolved a prokaryotic organelle; a membrane-bounded compartment with a specific cellular function: energy metabolism.

## Introduction

Anammox bacteria perform anaerobic ammonium oxidation (anammox) to dinitrogen gas and are applied for removal of ammonium from wastewater. They are also important in nature where they contribute significantly to oceanic nitrogen loss (Devol, 2003; Kuypers *et al*., 2003; 2005; Arrigo, 2005; Brandes *et al*., 2007). Anammox bacteria belong to the phylum *Planctomycetes*. One of the unique properties of species within this phylum is that they do not conform to the prokaryotic cell plan: their cells are compartmentalized and are proposed to lack a typical bacterial cell wall (Fuerst, 1995; Lindsay *et al*., 2001). The planctomycete compartmentalization is in some cases complex but always involves a single intracytoplasmic membrane defining a major cell compartment. Electron microscopy observations, chemical analysis, genome sequencing and resistance to beta-lactam and other cell wall-targeting antibiotics have revealed that planctomycetes lack the otherwise universal bacterial cell wall polymer peptidoglycan (König *et al*., 1984; Liesack *et al*., 1986; Stackebrandt *et al*., 1986; Fuerst, 1995) and an outer membrane typical of Gram-negative bacteria. The outermost membrane in planctomycetes has been defined as the cytoplasmic membrane based on the detection of RNA directly on its inner side by immunogold labelling and the position of the membrane and membrane surfaces relative to the cell wall visualized in thin sections of cryofixed and freeze-substituted cells and in freeze-fracture replicas (Lindsay *et al*., 1997; 2001;). The other, innermost, membrane has been defined as an intracytoplasmic membrane as it is within the cytoplasm and topologically inside the space bounded by the cytoplasmic membrane. The two compartments that are thus formed have been named ‘paryphoplasm’– the outermost compartment between cytoplasmic and intracytoplasmic membrane – and ‘riboplasm’– the innermost compartment inside the space bounded by the intracytoplasmic membrane. The riboplasm is the standard cytoplasmic compartment containing ribosomes and in most cases the nucleoid.

**Fig. 1:**
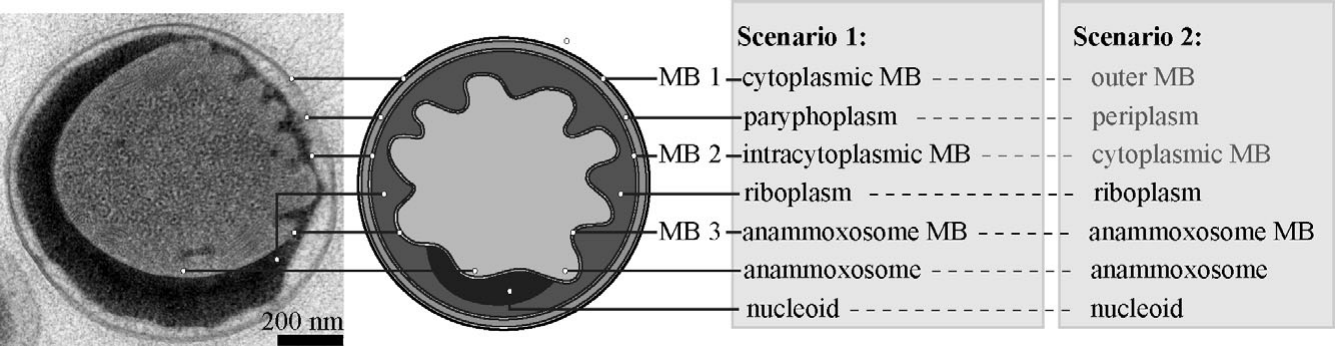
Electron micrograph (left) of an anammox bacterium and schematic overview (right) of the different scenarios regarding its cell plan. MB, membrane. Modified from van Niftrik *et al*. (2008b).

Anammox planctomycetes have an additional intracytoplasmic membrane compared with the standard planctomycete cell plan described above. The anammox cell is thus divided into three compartments by three individual bilayer membranes, from out- to inside; the paryphoplasm, riboplasm and anammoxosome compartment (Fig. 1). Anammox bacteria also contain unusual membrane lipids with ladderane moieties (linearly concatenated cyclobutane rings). These ladderane lipids are major membrane lipids of anammox membranes and are hypothesized to make these membranes highly impermeable (Sinninghe Damsté*et al*., 2002; 2005;) and to provide structural integrity to the cell.

**Fig. 2:**
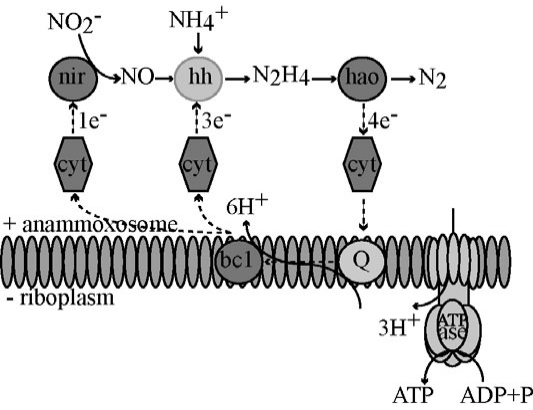
Postulated model for catabolic anammox reactions coupled over the anammoxosome membrane in anammox bacteria resulting in a proton-motive-force and subsequent ATP synthesis. bc1, cytochrome *bc1* complex; cyt, cytochrome; hao, hydrazine/hydroxylamine oxidoreductase; hh, hydrazine hydrolase; nir, nitrite reductase; Q, co-enzyme Q. Modified from Strous *et al*. (2006).

The anammoxosome compartment is hypothesized to be the site where the anammox catabolism takes place. Based on the genome of the anammox bacterium ‘*Candidatus* Kuenenia stuttgartiensis’, a biochemical model (Fig. 2) has been proposed where the anammox reaction is catalysed by several cytochrome *c* proteins (Strous *et al*., 2006). In this model, nitrite is first reduced to nitric oxide, then nitric oxide is combined with ammonium to form hydrazine and finally hydrazine is oxidized to dinitrogen gas. The four electrons derived from the oxidation of hydrazine to dinitrogen gas are fed into a respiratory chain at the level of ubiquinone via soluble cytochrome *c* electron carriers and a hypothetical cytochrome *c*: ubiquinone oxidoreductase (Cirpus *et al*., 2005; Huston *et al*., 2007). The anammox reaction thus establishes a proton gradient by the translocation of protons from the riboplasm to the anammoxosome, giving rise to a proton-motive-force. This proton-motive-force could then be used by membrane-bound ATPases to produce ATP. Experimental evidence that supports the proposed model is the immunogold localization of the enzyme hydrazine/hydroxylamine oxidoreductase (HAO) to the anammoxosome (Lindsay *et al*., 2001) and the demonstration that all, or almost all, cytochrome *c* proteins are located in the anammoxosome by cytochrome peroxidase staining (van Niftrik *et al*., 2008a). Furthermore, preliminary results from ^31^P nuclear magnetic resonance (NMR) spectroscopy showed the presence of two phosphate peaks in *K. stuttgartiensis* cells indicating an intracytoplasmic pH gradient (van der Star *et al*., 2010).

Some questions concerning the anammox cell plan remain. Although it has been assumed that anammox bacteria share the planctomycete cell plan, there are at least two possibilities with respect to the nature of the paryphoplasm (or region equivalent to the paryphoplasm) in anammox bacteria. As in other planctomycetes, the anammox paryphoplasm may represent a space between a true cytoplasmic membrane and an intracytoplasmic membrane (Fig. 1; scenario 1). On the other hand, the paryphoplasm might represent a space similar to the periplasm of Gram-negative bacteria if the outermost membrane of the cell is more like an outer membrane of a Gram-negative cell wall and the intracytoplasmic membrane is actually the cytoplasmic membrane (Fig. 1; scenario 2).

To explore such potential similarities to a Gram-negative cell plan, the genome of *K. stuttgartiensis* (Strous *et al*., 2006) was examined by comparative genomic analysis, which indicated that *K. stuttgartiensis* may be genetically capable of the biogenesis of a periplasm and outer membrane. First, a number of open reading frames (ORFs) were homologous to outer membrane porins. These porin homologues were absent in the genome of the planctomycete *Rhodopirellula baltica*. Second, the *K. stuttgartiensis* genome encoded the complete TonB system, a protein complex that relays energy from the cytoplasmic membrane to the outer membrane to drive a number of outer membrane receptors, five of which were also encoded in the genome. Third, *K. stuttgartiensis* encoded a number of typical three-component Gram-negative multidrug exporters, which consist of a cytoplasmic membrane, a periplasmic and an outer membrane subunit (‘gated porins’). Fourth, a partial peptidoglycan biogenesis pathway was encoded, including a number of penicillin-binding proteins. The only step not present in the peptidoglycan pathway of this bacterium was the ability to cross-link the glycan. With respect to all these four points, *R. baltica*, another planctomycete with a publicly available genome, contains hardly any genetic potential for Gram-negative cell wall structure or peptidoglycan synthesis. This might be consistent with the view that the paryphoplasm (Fig. 1; scenario 1) in *K. stuttgartiensis* may actually be more similar to a ‘regular’ periplasm (Fig. 1; scenario 2). However, the presence of these genes could also be a result of lateral gene transfer or be remainders of the evolutionary ancestor of anammox bacteria, which would then be a Gram-negative bacterium.

In contrast to the genomic evidence that could support the paryphoplasm being a periplasmic-like space, there is experimental evidence that supports the paryphoplasm being a cytoplasmic compartment with the cytoplasmic membrane on its outer side and the absence of a typical bacterial cell wall. First, neither peptidoglycan nor a typical outer membrane can be observed in transmission electron micrographs of all known species of anammox bacteria when examined after cryofixation and freeze-substitution or via classical chemical fixation (van Niftrik *et al*., 2008a,b;). Second, anammox bacteria and other non-anammox planctomycetes are prone to osmotic collapse under both hypotonic and hypertonic conditions (see Lindsay *et al*., 1995; 2001; van de Graaf *et al*., 1996), an indication that their structural integrity, normally derived from the presence of a cell wall, is not optimal. Third, the cell division ring of anammox bacteria is situated in the paryphoplasm compartment (van Niftrik *et al*., 2009). In general, the bacterial cell division ring is on the inside of, and closely opposed to, the cytoplasmic membrane indicating that the membrane on the outside of the paryphoplasm (Fig. 1; membrane 1) is the cytoplasmic membrane. Fourth, the apparent absence of cytochrome *c* proteins in the paryphoplasm as indicated by cytochrome peroxidase staining (van Niftrik *et al*., 2008a) supports the notion that this cannot be a typical periplasmic space analogous to that of Gram-negative bacteria.

A third scenario may reconcile the experimental and genomic evidence. In this scenario there is a periplasmic-like space outside the outermost membrane (Fig. 1; membrane 1), as proposed for some Archaea with S-layer walls (Küper *et al*., 2010) and for some Gram-positive bacteria (Zuber *et al*., 2008). This periplasmic-like space could be lost during sample preparation for electron microscopy.

The localization of anammox membrane-bound ATPases would resolve both the function of the anammoxosome and the location of the cytoplasmic membrane. If an ATPase was present on the innermost (anammoxosome) membrane (Fig. 1; membrane 3) this would strongly suggest that this compartment is indeed used for the generation of energy analogous to the function of mitochondria in Eukaryotes. The localization of an anammox ATPase to the outermost membrane of the anammox cell (Fig. 1; membrane 1) would indicate that this is indeed an energized, cytoplasmic membrane, and not an outer membrane typical of a Gram-negative cell wall, and that the middle anammox membrane (Fig. 1; membrane 2) is indeed an intracytoplasmic membrane as was initially proposed on structural grounds (Lindsay *et al*., 2001).

**Fig. 3:**
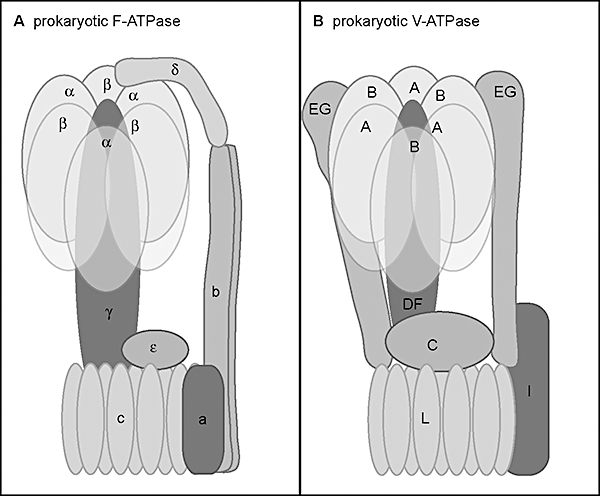
Schematic model of (A) a prokaryotic F-ATPase and (B) a prokaryotic V-ATPase. Assembled from Grüber *et al*. (2001), Lolkema *et al*. (2003), Yokoyama and Imamura (2005) and Lau and Rubinstein (2010).

Three different types of membrane-bound ATPases have been described: F-ATPase, A-ATPase and V-ATPase (Grüber *et al*., 2001; Grüber and Marshansky, 2008). V-ATPase (vacuolar-type) functions as an ATP-dependent proton-pump in eukaryotes (Drory and Nelson, 2006). F-ATPase and prokaryotic V-ATPase (the latter found in Bacteria and Archaea) can function both as ion-pump or as ATP synthase (Mitchell, 1961; Müller *et al*., 1999). The prokaryotic V-ATPase is also known as A-ATPase (archaeal-type) or V/A-ATPase (Grüber *et al*., 2001; Yokoyama and Imamura, 2005). Subunit composition can differ, but in general the prokaryotic V-ATPase consists of nine subunits and the bacterial F-ATPase consists of eight subunits (Stock *et al*., 2000; Lolkema *et al*., 2003) (Fig. 3). Membrane-bound ATPases contain two components: a membrane-bound, hydrophobic part (F_0_/A_0_/V_0_) containing the ion channel that is connected by a central stalk to the cytoplasmic, hydrophilic part (F_1_/A_1_/V_1_) containing the catalytic sites. The way F-ATPase functions is described by the binding change (Boyer, 1993; 1997;) and rotary mechanism (Stock *et al*., 2000). F_1_-ATPase contains three catalytic β subunits that perform ATP synthesis (or in some cases hydrolysis). F_0_-ATPase translocates protons (or sodium ions) across the membrane down the electrochemical gradient. In coupling proton transport to ATP synthesis, the F_0_ and F_1_ domains function as a pair of rotary motors linked by a common central rotor (γεc) and a peripheral stator (bδ). The multiple c-subunits (proteolipids) form a ring and rotation of this c-ring allows protons to be carried between two partial subunit-a channels that lead to opposite sites of the membrane. Each c-subunit contains two transmembrane helices that are connected by a cytoplasmic loop and one protonizable, carboxylate group (glutamate or aspartate; active carboxylate of *Escherichia coli* subunit c is Asp-61). The active carboxylate undergoes protonation/deprotonation cycles during proton transport and is located in helix 2 (Rastogi and Girvin, 1999). Either three or four protons need to be transported in sequence for a group of 12 c-subunits to move 120 degrees and promote the release of one ATP. Among the different membrane-bound ATPase types, the number of proteolipid transmembrane helices, and the number of proteolipid subunits per enzyme, differs.

Here, we identified and analysed four ATPase gene clusters in the *K. stuttgartiensis* genome. We show by transcriptomic, proteomic and immunoblot analyses that only one of these four ATPase gene clusters is likely to be expressed under the conditions investigated. Antiserum targeting this typical F-ATPase was used to locate this anammox membrane-bound ATPase in the anammox cell using immunogold localization. The typical F-ATPase was detected on all three anammox cell membranes but was predominantly present on both the innermost (anammoxosome) membrane and outermost membrane of the anammox cell. This indicates that the anammoxosome is used for the generation of ATP, probably vectorially from a proton-motive-force across the anammoxosome membrane itself, and is consistent with the identification of the outermost membrane of the anammox cell as the cytoplasmic membrane.

## Results

### ATPase gene clusters in the *K. stuttgartiensis* genome

**Fig. 4:**
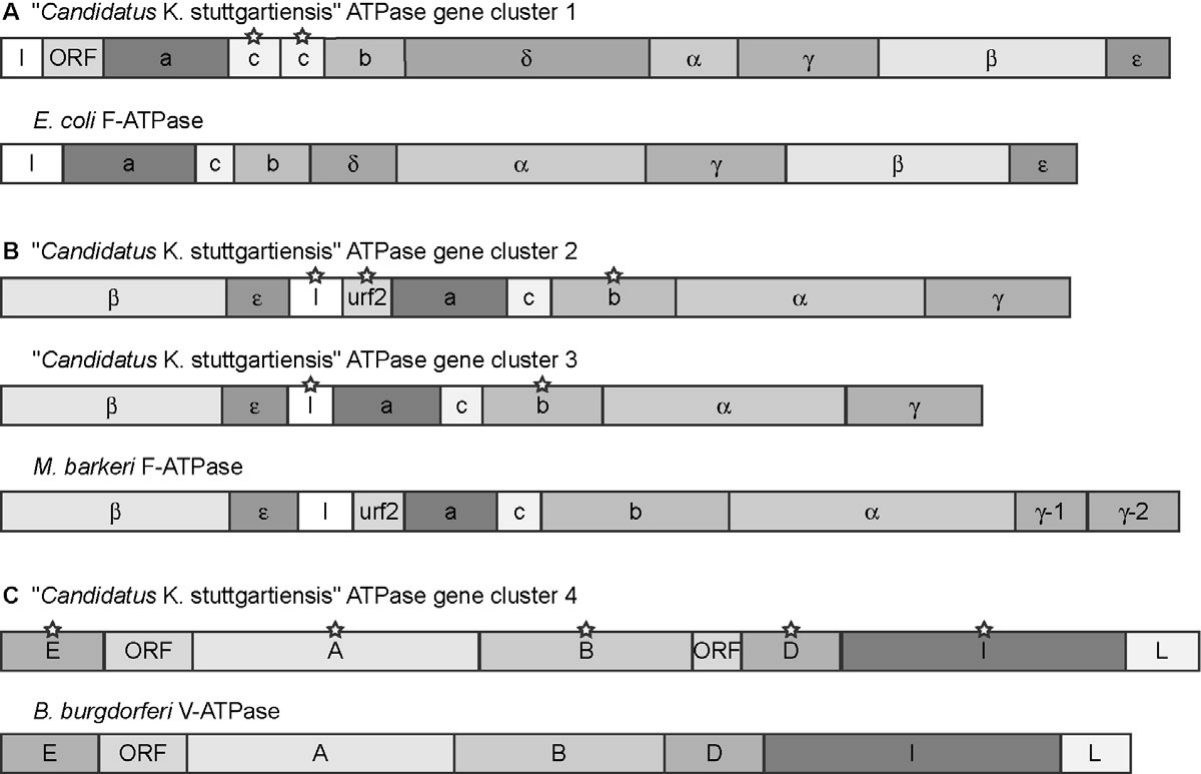
Comparison of the four ATPase gene clusters identified in the *K. stuttgartiensis* genome to their closest homologue. A. Gene cluster 1 (kuste3787–3796): F-ATPase similar to *E. coli* F-ATPase with the exception of an unknown ORF between gene I and subunit a and two ORFs encoding subunit c. B. Gene cluster 2 (kuste4592–4600) and 3 (kustc0572–0579): F-ATPases similar to *M. barkeri* F-ATPase. Gene cluster 3 lacks the unknown ORF, urf2, between gene I and subunit a. C. Gene cluster 4 (kuste3864–3871): V-ATPase similar to *B. burgdorferi* prokaryotic V-ATPase with the exception of an unknown ORF between subunit B and subunit D. Genes marked with a star are annotated as (conserved) hypothetical proteins. These genes have a sequence identity of over 20% to a protein with an undefined function or to a protein with a defined function but without similar sequence length. ORF, open reading frame.

Four putative gene clusters encoding membrane-bound ATPase complexes were identified in the genome assembly of *K. stuttgartiensis* (Strous *et al*., 2006) (Fig. 4). The syntheny (similarity of gene order) of gene cluster 1 was similar to the F-ATPase operon of *E. coli* with 23–69% sequence identities of individual orthologous genes to closest homologous genes with experimentally validated function. Note that in the databases where the *K. stuttgartiensis* genome has been deposited, the ORFs encoding for subunit b and subunit delta are translated in the wrong frame (3′5′−2 frame) as one ORF (kuste3792, unknown protein). Subunit b is actually encoded in the 5′3′+1 frame (169 aa, nucleotide position 1 430 441–1 430 947) and subunit delta is encoded in the 5′3′+2 frame (182 aa, nucleotide position 1 430 940–1 431 485). Putative gene clusters 2 and 3 were similar to each other and strongly resembled the atypical putative F-ATPase of *Methanosarcina barkeri* (Sumi *et al*., 1997) with 22–53% sequence identities of individual orthologous genes to closest homologous genes with experimentally validated function. The structure of gene cluster 4 resembled that of the *Borrelia burgdorferi* prokaryotic V-ATPase (Lolkema *et al*., 2003) with 30–72% sequence identities of individual orthologous genes to closest homologous genes (in this case all without experimentally validated function).

The protein sequences of the *K. stuttgartiensis* proteolipids were aligned using mega version 4 (Tamura *et al*., 2007) and were investigated for predicted transmembrane helices using tmhmm 2.0 (Krogh *et al*., 2001) and TMpred (Hofmann and Stoffel, 1993). The proteolipids of the F-ATPase gene clusters (subunit c, gene cluster 1–3) were aligned to the *E. coli* c-subunit (Fig. S1A). The *K. stuttgartiensis* c-subunits all consisted of two transmembrane helices and contained the subunit c signature motif. There was one discrepancy; subunit c from F-ATPase-2 contained glutamine (Q) instead of arginine (R) in the second position of the subunit c signature motif. The *K. stuttgartiensis* c-subunits all had glutamate (F-ATPase-1; both c-subunits Glu-58, F-ATPase-2 & -3; Glu-63) as the putative protonizable group. In F-ATPase-2 and -3 the protonizable group was not located in the predicted transmembrane helix 2 but instead was found one or two amino acids before. The proteolipid of the prokaryotic V-ATPase-4 gene cluster (subunit L) did not align very well to the *E. coli* proteolipid and was therefore aligned to its closest homolog; subunit L from *B. burgdorferi* (Fig. S1B). The prokaryotic V-ATPase-4 subunit L consisted of four transmembrane helices and helix 4 contained a putative skewed subunit c signature motif with Glu-137 as a putative protonizable group. In this motif, R-[NQ]-P was replaced by D-A-L. As in *B. burgdorferi*, no second protonizable group (D or E) was present in or near helix 2. Sequence analysis of the proteolipids indicated that the three F-ATPases are potentially capable of ATP synthesis. With respect to gene cluster organization, F-ATPase-1 contained the complete set of genes known to be part of the *E. coli* F-ATPase and therefore there is no reason why this gene cluster could not encode for a functional membrane-bound ATPase. F-ATPase-2 and -3 lacked a gene encoding the delta subunit that in F-ATPase connects the F_1_ and F_0_ static parts (Fig. 3), although it is unknown whether this would infer dysfunctionality. The prokaryotic V-ATPase-4 gene cluster contained both catalytic subunits (B and A), the proteolipid (L), ion channel (I), part of the rotor (D) and part of the stator (E), indicating that it could encode for a functional membrane-bound ATPase.

### *K. stuttgartiensis* transcriptomic and proteomic analysis

To investigate the expression of the four ATPase gene clusters identified in the genome, the transcriptome and proteome of *K. stuttgartiensis* were analysed for the presence of mRNA or detected peptides respectively (Table 1). The analysis showed that F-ATPase-1, the typical ATP synthase, was detected at high levels in both the transcriptome and proteome. This indicates that this ATPase gene cluster most likely encodes a functional membrane-bound ATPase in *K. stuttgartiensis*. Both atypical F-ATPases-2 and -3 were not detected in the proteome and showed very low transcription levels (relative expression < 1.0), making it unlikely that these two ATPase gene clusters function in energy generation in *K. stuttgartiensis* cells at least under the cultivation conditions of this study. For the prokaryotic V-ATPase-4 the catalytic subunits (A and B) were detected in the transcriptome (relative expression > 1.0) and one peptide (subunit A) was detected in the proteome. More data will be needed to make a functional assignment possible for the prokaryotic V-ATPase.

**Table 1:** Presence of mRNA (transcriptome) and peptides (proteome) from the four ATPase gene clusters present in the genome of *K. stuttgartiensis*.

Genome				Transcriptome		Proteome	
ORF	Putative ATPase subunit	aa	nt	# unique reads detected	Relative expression	# unique peptides detected	Gene coverage (%)
F-ATPase-1							
kuste3787	I	84	255	50	0.9	ND	NA
kuste3788	*Unknown*	124	375	136	1.6	ND	NA
kuste3789	a	257	774	656	3.7	2	25
kuste3790	c	108	327	204	2.8	ND	NA
kuste3791	c	90	273	317	5.1	1	25
kuste3792	b	169	510	882	7.6	ND	NA
kuste3792	Delta	182	549	1013	8.1	ND	NA
kuste3793	Alpha	504	1515	1881	5.5	23	66
kuste3794	Gamma	290	873	415	2.1	8	29
kuste3795	Beta	471	1416	1956	6.1	24	60
kuste3796	Epsilon	134	405	413	4.5	3	27
F-ATPase-2							
kuste4592	Beta	462	1389	74	0.2	ND	NA
kuste4593	Epsilon	130	393	16	0.2	ND	NA
kuste4594	I	111	336	31	0.4	ND	NA
kuste4595	*Unknown*	101	306	18	0.3	ND	NA
kuste4596	a	236	711	68	0.4	ND	NA
kuste4597	c	93	282	15	0.2	ND	NA
kuste4598	b	255	768	21	0.1	ND	NA
kuste4599	Alpha	513	1542	101	0.3	ND	NA
kuste4600	Gamma	299	900	67	0.3	ND	NA
F-ATPase-3							
kustc0572	Beta	454	1365	177	0.6	ND	NA
kustc0573	Epsilon	134	405	12	0.1	ND	NA
kustc0574	I	92	279	32	0.5	ND	NA
kustc0575	a	221	666	39	0.3	ND	NA
kustc0576	c	88	267	34	0.6	ND	NA
kustc0577	b	246	741	35	0.2	ND	NA
kustc0578	Alpha	498	1497	78	0.2	ND	NA
kustc0579	Gamma	279	840	16	0.1	ND	NA
V-ATPase-4							
kuste3864	E	210	633	99	0.7	ND	NA
kuste3865	*Unknown*	180	543	74	0.6	ND	NA
kuste3866	A	589	1770	531	1.3	1	2
kuste3867	B	437	1314	503	1.7	ND	NA
kuste3868	*Unknown*	99	300	36	0.5	ND	NA
kuste3869	D	202	609	21	0.2	ND	NA
kuste3870	I	583	1752	218	0.6	ND	NA
kuste3871	L	152	459	66	0.6	ND	NA

ORF, open reading frame; aa, amino acids; nt, nucleotides; ND, not detected; NA, not applicable. The relative expression (transcriptome) is calculated as: [# unique reads detected * length of the reads (75 nt)/gene length (nt)]/coverage of the transcriptome (= 17). The gene coverage (proteome) is calculated as: (# unique peptides detected/# unique peptides predicted) * 100%.

### Heterologous expression of the catalytic subunits of the four *K. stuttgartiensis* ATPase gene clusters and subsequent antibody production

**Fig. 5:**
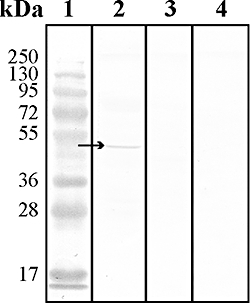
Immunoblot analysis of the antiserum directed at the catalytic beta subunit of the F-ATPase-1 gene cluster found in the *K. stuttgartiensis* genome. Lane 1: Marker (PageRuler™ Prestained Protein Ladder Plus, Fermentas); lane 2: incubation with anti-F-ATPase-1; lane 3: incubation with the pre-immune serum; lane 4: incubation with only the secondary antibody. Arrow: expected target size (52 kDa).

Parts of the catalytic beta (F-ATPases) and A (prokaryotic V-ATPase) subunits (Table S1) were cloned and expressed in *E. coli*. The purified protein fragments were used to raise antibodies in rabbit. All four antisera hybridized to the associated heterologous protein on dot blots (data not shown). The antisera were subsequently used to test for the presence of their target proteins in *K. stuttgartiensis* cell-free extract using SDS-polyacrylamide gel electrophoresis (SDS-PAGE) and immunoblot analysis. The antiserum targeting F-ATPase-1 showed a band at the expected size (52 kDa) that was absent in both negative controls (incubation with pre-immune serum and incubation with only the secondary antibody) (Fig. 5). Incubation with the other antisera (anti-F-ATPase-2, -F-ATPase-3 and -V-ATPase-4) did not result in the detection of a protein of the expected size, nor of any other specific bands (data not shown). These results were consistent with the high levels of transcription and expression observed for F-ATPase-1 and the undetectable or at least very low levels of transcription and expression of the other membrane-bound ATPases under the applied growth conditions.

Anti-F-ATPase-1 was further tested for its specificity and affinity using immunofluorescence analysis of formaldehyde-fixed *K. stuttgartiensis* cells. This analysis showed that the antiserum specifically bound to whole *K. stuttgartiensis* cells (Fig. S2) while the pre-immune serum had only a very faint background at the same exposure time. This indicated that this antiserum was suitable for immunogold localization to identify the cellular location of this membrane-bound ATPase in the anammox cell.

### Immunogold localization of F-ATPase-1 in *K. stuttgartiensis*

**Fig. 6:**
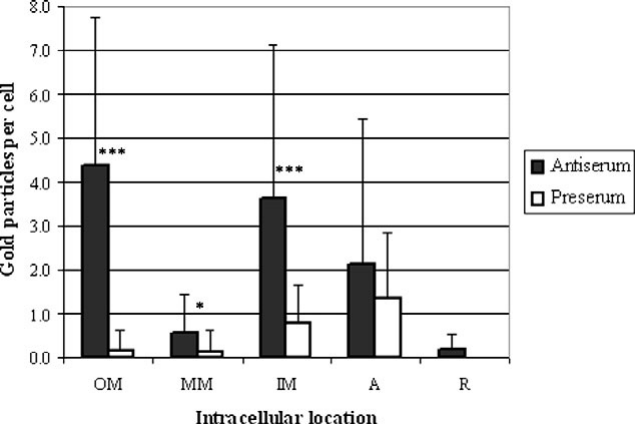
Immunogold labelling distribution (average gold particles per cell per location) in *K. stuttgartiensis* using the antiserum directed at F-ATPase-1 and its pre-immune serum. All three membranes of the anammox cell are significantly labelled compared with the incubation with the pre-immune serum (the negative control). OM, outermost membrane (membrane 1); MM, middle membrane (membrane 2); IM, innermost (anammoxosome) membrane (membrane 3); A, anammoxosome; R, riboplasm; ***, extremely statistically significant; *, statistically significant.

**Fig. 7:**
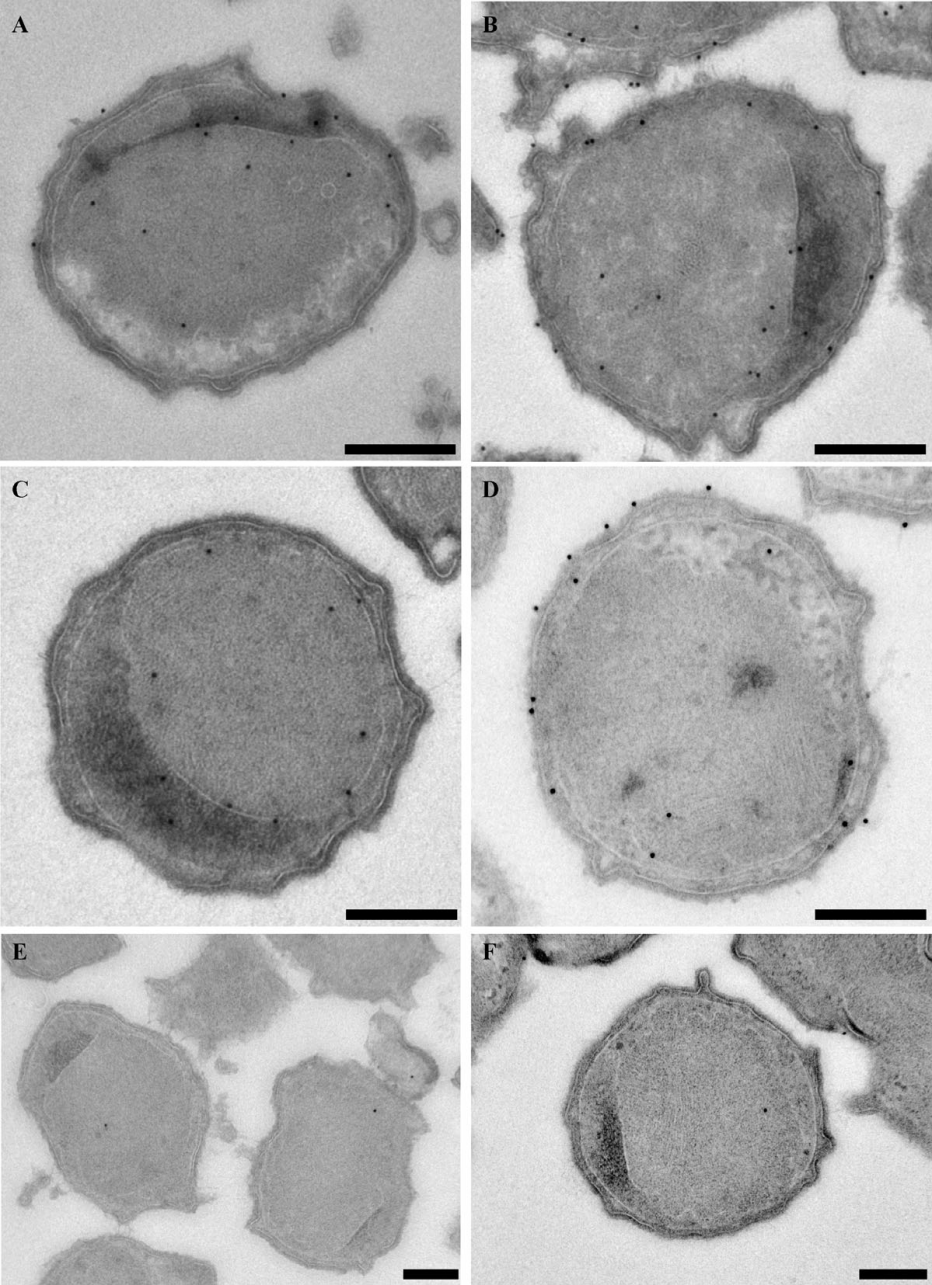
A–D. Immunogold localization of the antiserum directed at the catalytic beta subunit of the F-ATPase-1 gene cluster localizes this ATPase to all anammox membranes; the outermost membrane (membrane 1), middle membrane (membrane 2) and innermost (anammoxosome) membrane (membrane 3) in *K. stuttgartiensis* rehydrated cryosections. See Fig. S3 for annotation of specific gold labels. E and F. Negative control: incubation with the pre-immune serum instead of the antiserum. Scale bars: 250 nm.

The antiserum targeting F-ATPase-1 was used in immunogold localization to determine its location in anammox cells prepared via the rehydration method (van Donselaar *et al*., 2007) and subsequent cryosectioning. All three membranes of the anammox cell, the outermost membrane (membrane 1), middle membrane (membrane 2) and innermost (anammoxosome) membrane (membrane 3), which appear as white trilamina in the cryosections, were significantly labelled (Figs 6 and 7 and Fig. S3) compared with the incubation with the pre-immune serum (the negative control). There was some background labelling in the anammoxosome and riboplasm but this was not significantly more in the incubation with the antiserum than in the incubation with the pre-immune serum. Since gold particles that could be attributed to more than one membrane were not taken into account, the labelling of the membranes is even underestimated. There was significantly more labelling on the outermost membrane and the innermost (anammoxosome) membrane compared with the middle membrane while there was no significant difference between the labelling of the outermost membrane and the innermost (anammoxosome) membrane. From these results, it can be concluded that the F-ATPase-1 is predominantly located on the innermost (anammoxosome) membrane (membrane 3) and the outermost membrane (membrane 1) and to a lesser extent on the middle membrane (membrane 2).

## Discussion

In this study, the intracellular location of membrane-bound ATPases in the anammox bacterium *K. stuttgartiensis* was addressed to investigate the hypothesis that the anammoxosome compartment and its membrane are used for the generation of energy and to identify the cytoplasmic membrane. Four putative ATPase gene clusters (F-ATPase-1, -2, -3, and prokaryotic V-ATPase-4) were identified in the *K. stuttgartiensis* genome. Transcriptomic, proteomic and immunoblot analyses with antisera directed at the catalytic subunits indicated that F-ATPase-1 was the most significant membrane-bound ATPase under the growth conditions investigated. Immunogold localization showed that this F-ATPase was predominantly present on the innermost (anammoxosome) membrane and outermost membrane of the anammox cell and to a lesser extent on the middle membrane.

### Four ATPase gene clusters in the *K. stuttgartiensis* genome

The genome of *K. stuttgartiensis* encodes four putative ATPase gene clusters: one typical F-ATPase (F-ATPase-1), two atypical F-ATPases (F-ATPase-2 and -3) lacking the delta subunit and a prokaryotic V-ATPase (V-ATPase-4).

F-ATPase-1 resembled most the typical F-ATPase from *E. coli* but also the F-ATPase from *Acetobacterium woodii* (Müller *et al*., 2005). The *K. stuttgartiensis* transcriptome showed a high relative expression (ranging from 0.9–8.1) of all genes in the F-ATPase-1 gene cluster and in the proteome, peptides of six of the 11 genes were detected (Table 1). Further, the antiserum directed at the catalytic beta subunit specifically detected a protein of the expected size in immunoblot analysis (Fig. 5) and hybridized specifically to formaldehyde-fixed *K. stuttgartiensis* cells in immunofluorescence (Fig. S2). In conclusion, the transcriptome, proteome, immunoblot and immunofluorescence data indicated that the F-ATPase-1 gene cluster encodes the major functional membrane-bound ATPase in *K. stuttgartiensis*.

The gene cluster structure of F-ATPase-2 and F-ATPase-3 strongly resembled that of the atypical *M. barkeri* F-ATPase (Fig. 4) (Sumi *et al*., 1997). These gene clusters lacked a gene encoding the delta subunit that in F-ATPase connects the F_1_ and F_0_ static parts (Fig. 3). Without this subunit, the ATPase enzyme would probably be less stable. Therefore, if these gene clusters are expressed they would have to acquire the delta subunit from somewhere else, which would have to be the F-ATPase-1 gene cluster. Another possibility could be that the extended version of their b-subunit (255 aa and 246 aa compared with 169 aa in F-ATPase-1, Table 1) has functionally replaced the delta subunit. In *M. barkeri* it is doubtful whether this F-ATPase is functional because the amino acid sequences of the genes are so deviated from the typical F-ATPase and also because mRNA and protein products cannot be detected (Sumi *et al*., 1997). A functional F-ATPase in *M. barkeri* would be quite unusual anyway since Archaea (including *M. barkeri*) typically have prokaryotic V-ATPases and not F-ATPases. The transcriptome of *K. stuttgartiensis* showed a very low relative expression level (< 1.0) for both ATPase gene clusters and no peptides were detected in the proteome (Table 1). Antibodies generated against the catalytic subunits also could not detect their target proteins in *K. stuttgartiensis* cell-free extract under the cultivation conditions of this study. In conclusion, the predicted instability of both enzymes by the absence of the delta subunit, the very low expression level in the transcriptome and absence of peptides in the proteome and the inability of the antisera to detect their target proteins in cell-free extract make it very unlikely that these two gene clusters encode functional F-ATPases in *K. stuttgartiensis*. Perhaps these two gene clusters are cryptic, were acquired through lateral gene transfer and were duplicated in a subsequent rearrangement event.

The gene cluster structure of V-ATPase-4 resembled most that of *B. burgdorferi* prokaryotic V-ATPase (Fig. 4). These two gene clusters, and also the chlamydial prokaryotic V-ATPase gene clusters (Lolkema *et al*., 2003), do not contain homologues of subunits C and F (part of the central stalk), and G (part of the stator) (Fig. 3). It was not clear from the transcriptome and proteome whether gene cluster V-ATPase-4 could encode for a functional membrane-bound ATPase. Only the catalytic subunits (A and B) were detected, at a low level, in the transcriptome and only one peptide (subunit A) was detected in the proteome. Further, the antiserum targeting the catalytic subunit A could not detect its target protein in *K. stuttgartiensis* cell-free extract. In conclusion, either the V-ATPase-4 gene cluster is not expressed as a functional membrane-bound ATPase under the cultivation conditions of this study or it is expressed at such low levels that it is difficult to detect with the current methods.

### The intracellular location of F-ATPase-1 in *K. stuttgartiensis* cells

All data indicated that the F-ATPase-1 gene cluster encoded a functional membrane-bound ATPase capable of ATP synthesis and that the other ATPase gene clusters were most likely not expressed in the cells used in this study. Therefore, only the antiserum targeting F-ATPase-1 was used in immunogold localization to determine its location in anammox cells prepared via the rehydration method and cryosectioning (van Donselaar *et al*., 2007). Note that, even if the atypical F-ATPase-2 and -3 could be expressed, cross-reactivity with the antiserum targeting the beta subunit of F-ATPase-1 was not to be expected since the antibody target of F-ATPase-1 showed only a 37% protein sequence identity with the beta subunits of the atypical F-ATPase-2 and -3. Anti-F-ATPase-1 located the F-ATPase to all three anammox membranes (Figs 6 and 7 and Fig. S3). The labelling was equally high on both the innermost (anammoxosome) membrane (membrane 3) and outermost membrane (membrane 1). Further, both of these membranes were significantly more densely labelled than the middle membrane (membrane 2) although the labelling on the middle membrane was still significantly higher than in the negative control. The immunogold labelling of the anammox membranes is even underestimated. First, gold particles that could be allocated to more than one membrane (i.e. within 25 nm distance from more than one membrane) were not taken into account. Second, labelling in the anammoxosome compartment could still be real innermost (anammoxosome) membrane labelling considering the high curvature of this anammoxosome membrane as visualized by electron tomography (van Niftrik *et al*., 2008b). These results indicate that the F-ATPase-1 is predominantly present on the innermost (anammoxosome) membrane and outermost membrane and to a lesser extent on the middle membrane and that thus all three membranes are energized and within the essential cell boundary. This is not consistent with the preliminary results using ^31^P NMR that indicated the presence of two pH peaks and thus most likely two energized membranes (van der Star *et al*., 2010). It is also inconsistent with the absence of genes encoding a putative nucleotide transporter in the genome of *K. stuttgartiensis.* Such a transporter would be necessary in a scenario with three energized membranes. Because most label was found on the anammoxosome and outer membranes we conclude that these membranes most likely have a proton-motive-force. Because the cytochromes are located inside the anammoxosome, F-ATPase-1 most likely produces ATP on this membrane. This ATP is then hydrolysed again by F-ATPase-1 on the outermost membrane. There are two explanations for the presence of F-ATPase in the middle membrane: it is the apo-complex *en route* to its final destination or it is functional, which we cannot explain at this moment.

In any case, the results so far indicate that the outermost membrane (membrane 1) is the cytoplasmic membrane as was initially proposed (Fig. 1, scenario 1) (Lindsay *et al*., 2001). To some extent, the ultrastructure and ATPase immunolabelling of the outermost membrane of *K. stuttgartiensis* resembles that of the Crenarchaeon *Ignicoccus hospitalis* (Küper *et al*., 2010). *I. hospitalis* also has a compartmentalized cell plan and an energized outermost membrane. However, in the *I. hospitalis* case, the outermost compartment has been defined as a periplasmic-like space based on its low electron density in transmission electron micrographs, which suggested the apparent absence of ribosomes and DNA. Consequently, when the outermost membrane was found to be energized, it was concluded that *I. hospitalis* has an energized ‘outer membrane’ (although this membrane is structurally not similar to the outer membrane of Gram-negative bacteria) and ATP synthesis in the ‘periplasm’. In a way, this resembles the *K. stuttgartiensis* case but in our case the compartments and membranes are defined differently. This raises the question what defines the cytoplasm, periplasm, cytoplasmic membrane and outer membrane?

The genetic potential found in the *K. stuttgartiensis* genome for producing a Gram-negative-like cell wall could be a cryptic result of lateral gene transfer or be a remainder of the evolutionary ancestor of anammox bacteria, which would then be a Gram-negative bacterium. However, another possibility is that there is indeed a Gram-negative-like cell wall in anammox bacteria but that this is lost during sample preparation for electron microscopy. Anammox bacteria certainly do not live in osmotically protected areas like the pathogenic cell wall-less mycoplasma species, so are in need of some form of structural integrity. Other planctomycetes possess proteinaceous cell walls and their cells show considerable structural integrity, even able in some cases to withstand treatment with 10% SDS at 100°C (König *et al*., 1984; Liesack *et al*., 1986), conditions under which Gram-negative cell walls and especially outer membranes would be expected to disintegrate. Perhaps in the anammox case, the ladderane lipids provide the structural integrity that most other bacteria derive from their cell wall. These lipids have been predominantly found in the anammoxosome membrane but also occur in one or both of the other two anammox cell membranes (Sinninghe Damsté*et al*., 2002). Alternatively, we may in fact lack some structural information and there may be yet another layer to the anammox cell. There is indeed some evidence of a regular protein surface layer (S-layer) lattice in *K. stuttgartiensis* from freeze-fracture replicas (Fuerst *et al*., 2006), but such an S-layer, if comparable to those protein or glycoprotein lattices of other bacterial and of archaeal cell walls, is unlikely to possess membrane properties. However, in Archaea, which often do not contain other cell wall components besides S-layers, S-layers are proposed to maintain cell shape and can be viewed as exoskeletons that contribute to mechanical and osmotic cell stabilization (Engelhardt, 2007). Perhaps in the anammox bacteria where, as in the Archaea, also no other cell wall components have been found, structural integrity is also derived from an S-layer lattice. Even though the complete loss of additional cell layers seems unlikely since many different fixation procedures have been applied to anammox bacteria (van Niftrik *et al*., 2008b), the presence of an outer membrane, exo- (S-layer) or endoskeleton (cytoskeleton) is currently under investigation by imaging anammox bacteria in their near-native state with cryoelectron microscopy.

The localization of an F-ATPase to the anammoxosome membrane shows that this compartment is indeed used for the generation of energy analogous to the function of mitochondria in Eukaryotes. With its curved membrane to maximize the membrane area available for the catabolic processes to take place (van Niftrik *et al*., 2008b), the localization of the key anammox protein HAO (Lindsay *et al*., 2001) and cytochrome *c* proteins (van Niftrik *et al*., 2008a) to the anammoxosome compartment, and the presence of an ATP synthase on the anammoxosome membrane established here, it seems that anammox bacteria may indeed have evolved a bacterial organelle: a separate membrane-bounded compartment with a specific function inside the cell: energy metabolism.

## Experimental procedures

### *Kuenenia stuttgartiensis* cells

Samples containing an 80% enrichment culture of *K. stuttgartiensis* were taken from a 2 l sequencing batch reactor or a 15 l continuous reactor (modified from Strous *et al*., 1998).

### Genome analysis

*Kuenenia stuttgartiensis* ORFs encoding putative membrane-bound ATPase subunits were blasted (blastp) against the NCBI protein database (http://www.ncbi.nlm.nih.gov/BLAST/). Putative homologues were subsequently aligned using the PIR pairwise alignment tool (http://pir.georgetown.edu/pirwww/search/pairwise.shtml).

### Transcriptomic analysis

*Kuenenia stuttgartiensis* RNA was extracted using the Ribopure Bacteria Kit according to the manufacturer's instructions from Ambion (Foster City, CA, USA). First-strand cDNA was synthesized with random primers using the RevertAid™ H Minus First Strand cDNA Synthesis Kit, and the second strand was synthesized using DNA polymerase and manufacturer's instructions (Fermentas, Vilnius, Lithuania). The Solexa reads (3.5 million) were mapped using the CLC Workbench software version 3.7.1 (http://www.clcbio.com). The program reports expression as Reads Per Kilobase of exon model per Million mapped reads (RPKM).

### Proteomic analysis

*Kuenenia stuttgartiensis* single-cell suspensions were harvested by centrifugation. The cell pellet was resuspended in one volume of 20 mM potassium phosphate buffer pH 8, and passed three times through a French pressure cell operated at 138 MPa. After centrifugation for 15 min at 1700 *g* at 4°C, a cell-free fraction was obtained as clarified supernatant. A sample of the cell-free extract was denatured by incubation with 60 mM Tris-HCl buffer pH 8 containing 5% β-mercaptoethanol, 2% sodium dodecyl sulphate (SDS) and 25% glycerol for 5 min at 100°C. SDS-PAGE was performed in 10% or 6% slab gels in 375 mM Tris hydrochlorideglycine buffer, pH 8.8, with approximately 50 µg of protein per lane. After separation of proteins and staining with colloidal Coomassie Brilliant Blue, the gel lane was cut into four slices, and each gel slice was destained with three cycles of washing with successively 50 mM ammonium bicarbonate and 50% acetonitrile (ACN). Sample analysis by LC-MS/MS, including protein reduction, alkylation and digestion, was performed as described previously (Ettwig *et al*., 2010).

### Antibody production

For the antisera directed against the catalytic beta (F-ATPase-1, -2 and -3) or A subunits (V-ATPase-4), we expressed and purified parts of the *K. stuttgartiensis* kuste3795 (ATPase gene cluster 1), kuste4592 (ATPase gene cluster 2), kustc0572 (ATPase gene cluster 3) and kuste3866 (ATPase gene cluster 4) ORFs in *E. coli* as described previously (Harhangi *et al*., 2002), with the following changes. Primers were designed on the sequences (Table S1). All forward primers started on nucleotide position 1 of the ORF. For directional cloning, restriction sites were included in all primers. Further, stop codons were introduced in the reverse primers so as to express only an N-terminal His-tag. As an expression vector, pET30a-c(+) (Novagen, Darmstadt, Germany) was used, and as the host, Rosetta cells (Novagen, Darmstadt, Germany). The heterologous expressed protein fragments were purified using the nickel-nitrilotriacetic acid (Ni/NTA) protein purification system (Qiagen, Venlo, the Netherlands) with an 8 M urea, 100 mM NaH_2_PO_4_, 10 mM Tris-HCl buffer at different pH levels (6.3, 6.1, 5.9, 5.7, 5.5, 5.0 and 4.5) and imidazole concentrations (300, 250, 200, 150, 100, 50 and 20 mM). The identities of the expressed protein fragments were verified by MALDI-TOF MS peptide mass fingerprinting of a tryptic digest of the Ni-NTA purified protein (Harhangi *et al*., 2002). The expressed protein fragments were used to immunize rabbits in a 3-month immunization protocol (SEQLAB Sequencing Laboratories Göttingen GmbH, Göttingen, Germany). The antisera (anti-F-ATPase-1, -2, -3 and anti-V-ATPase-4) were then used as the primary antiserum (polyclonal, crude serum) in immunoblot analysis and (anti-F-ATPase-1) immunofluorescence and immunogold localization as described below.

### SDS-PAGE and immunoblot analysis

*Kuenenia stuttgartiensis* proteins (cell-free extract prepared using French press) were separated (50 µg of protein per lane) on a 10% SDS-PAGE gel and transferred to a cellulose-nitrate membrane (Schleicher & Schuell GmbH, Dassel, Germany) with the semi-dry transfer cell blotting system (Bio-Rad, Veenendaal, the Netherlands). Blotting was performed at 50 mA for 3 h with a transfer buffer that consisted of 25 mM Tris, 192 mM glycine and 20% methanol. After blotting, the blot was dried and stored at 4°C until further use.

Blots stored at 4°C were washed in MilliQ water for 30 min and incubated in blocking buffer; 1% bovine serum albumin (BSA) in Tris-buffered saline (TBS; 10 mM Tris-HCl, 0.9% NaCl, pH 7.4) for 1 h. The blot was then incubated for 1 h in either blocking buffer or rabbit pre-immune serum diluted 500-fold in blocking buffer as the negative controls or primary antiserum diluted 500-fold in blocking buffer. The blot was washed three times for 10 min in TBS containing 0.05% Tween20 and incubated for 1 h in monoclonal mouse anti-rabbit IgG alkaline phosphatase conjugate (Sigma, Zwijndrecht, the Netherlands) diluted 150.000-fold in blocking buffer. The blot was washed two times for 10 min in TBS containing 0.05% Tween20 and three times for 10 min in TBS. The blot was incubated with the BCIP/NBT Liquid Substrate System (Sigma, Zwijndrecht, the Netherlands) for 8 min, rinsed in excess amounts of MilliQ water and dried. All blots were scanned with the same settings.

### Immunofluorescence analysis

*Kuenenia stuttgartiensis* cells were washed with phosphate-buffered saline (PBS; 0.1 M phosphate, 0.137 M NaCl, 2.7 mM KCl, pH 7.4) and fixed in 3% formaldehyde in PBS for 120 min while rotating at 4°C. The cells were washed again with PBS, resuspended in 1:1 ethanol : PBS and stored at −20°C until further use.

Formaldehyde-fixed cells were transferred to 0.075% gelatine, 0.01% chromium coated, six-well diagnostic microscope slides (Menzel GmbH & Co. KG, Braunschweig, Germany) and allowed to air dry. Next, the cells were permeabilized by a 10 min incubation with 0.5% Triton X-100 in TBS and washed three times with TBS. A-specific binding sites were blocked by a 30 min incubation with blocking buffer (1% BSA in TBS) in a moist incubation chamber at room temperature after which the blocking buffer was removed and the cells were allowed to air dry. Cells were incubated at room temperature for 1 h with primary antiserum diluted 500- or 1000-fold in blocking buffer in a moist incubation chamber. Each specific well was washed twice with 0.1% BSA in TBS after which the entire slide was washed three times for 10 min in 100 ml of 0.1% BSA in TBS by shaking rigorously. Cells were allowed to air dry and were then incubated for 1 h with the secondary antibody, Cy-3-labelled sheep anti-rabbit IgG (Sigma, Zwijndrecht, the Netherlands), diluted 100-fold in blocking buffer at room temperature in a moist incubation chamber in the dark. Each specific well was washed twice with TBS after which the entire slide was washed three times for 10 min in the dark in 100 ml of TBS by shaking rigorously. The cells were allowed to air dry, Vectashield mounting medium with 4,6-diamidino-2-phenylindole (DAPI) (Vector Laboratories, Burlingame, USA) was added to each well, the coverslip was sealed with nail polish and the slides were stored in the dark at 4°C until further investigation.

Several control treatments were used. All control treatments were performed on *K. stuttgartiensis* cells unless stated otherwise, substituting primary antiserum and/or secondary antibody respectively. Four negative controls were performed: blocking buffer combined with blocking buffer, blocking buffer combined with secondary antibody, rabbit pre-immune serum combined with secondary antibody and rabbit anti-*Nitrosomonas* sp. combined with secondary antibody. Two positive controls were performed: rabbit anti-anammox hydrazine/hydroxylamine oxidoreductase (Schalk *et al*., 2000) combined with secondary antibody and rabbit anti-*Nitrosomonas* sp. combined with secondary antibody on *Nitrosomonas* sp. cells.

Cells were investigated at 1.000× magnification with a Zeiss Axioplan 2 imaging epifluorescence microscope (Carl Zeiss B.V., Sliedrecht, the Netherlands).

### Sample preparation for immunogold localization: cryofixation, freeze-substitution and cryosectioning (rehydration method) *(**van Donselaar* et al.*, 2007**)*

Small aggregates of *K. stuttgartiensis* cells were cryofixed by high-pressure freezing and freeze-substituted in acetone containing 0.5% glutaraldehyde and 1% H_2_O as described previously (van Niftrik *et al*., 2008b). After freeze-substitution, fixation was continued for 60 min on ice. Samples were rehydrated in a graded acetone series on ice: 95%, 90%, 80% and 70% acetone in water containing 0.5% glutaraldehyde, then 50 and 30% acetone in PHEM buffer (60 mM PIPES, 25 mM HEPES, 10 mM EGTA, 2 mM MgCl_2_, pH 6.9) containing 0.5% glutaraldehyde, and finally 0.5% glutaraldehyde in PHEM buffer. Samples were rinsed in PHEM buffer and embedded in 12% gelatin in PHEM buffer. The gelatin-embedded cells were cut into small cubes (1–2 mm^3^) under the stereo microscope, infiltrated overnight at 4°C with 2.3 M sucrose in PHEM buffer and frozen in liquid nitrogen.

Samples were cryosectioned using a cryoultramicrotome UC6/FC6 (Leica Microsystems, Vienna, Austria). Ultrathin cryosections (55 nm) were picked up with a drop of 1% methyl cellulose and 1.15 M sucrose in PHEM buffer and transferred to carbon-formvar-coated grids (copper, 100 mesh, hexagonal) for immunogold localization.

### Immunogold localization

Grids containing ultrathin cryosections of *K. stuttgartiensis* cells were washed for 30 min at 37°C with PBS, incubated for 10 min at room temperature on drops of PBS containing 20 mM glycine and blocked for 15 min on drops of PBS containing 1% BSA or 2% skim milk powder (Frema Reform, Germany). After blocking, the grids were incubated for 60 min with the primary antiserum diluted 50-fold in PBS containing 1% BSA or 2% skim milk powder and washed for 12 min on drops of PBS containing 0.1% BSA or 0.2% skim milk powder. Grids were incubated for 20 min with protein A coupled to 10 nm gold (PAG-10, CMC/UMC, Utrecht, the Netherlands), diluted 80-fold in PBS containing 1% BSA or 2% skim milk powder and washed for 14 min on drops of PBS. The cryosections on grids were fixed for 5 min with PBS containing 1% glutaraldehyde and washed for 10 min on drops of distilled water. Cryosections were post-stained for 5 min with 2% uranyl acetate in 0.15 M oxalic acid pH 7.4 and washed quickly on two drops of distilled waster and then on two drops of 1.8% methyl cellulose containing 0.4% aqueous uranyl acetate on ice. Finally, cryosections were embedded for 5 min in 1.8% methyl cellulose containing 0.4% aqueous uranyl acetate on ice after which they were air dried.

Several control treatments were performed. As a positive control, grids were incubated with rabbit anti-anammox hydrazine/hydroxylamine oxidoreductase (Schalk *et al*., 2000) as the primary antiserum. Negative controls were: incubation with pre-immune serum instead of primary antiserum, incubation with affinity-isolated rabbit anti-influenza haemagglutinin (anti-HA, H6908, Sigma, Zwijndrecht, the Netherlands) as a primary antiserum, and incubation with blocking buffer instead of primary antiserum. Further, PAG-10 was used instead of a secondary antibody (such as sheep anti-rabbit IgG) because PAG-10, unlike other secondary antibodies, gave no background in the negative control with only PAG-10. Also, we used 10 nm gold because there was sufficient labelling and it was not necessary to use smaller gold to prevent steric hindrance.

Grids containing ultrathin cryosections of *K. stuttgartiensis* cells were investigated at 80 kV in a transmission electron microscope (Tecnai12, FEI Company, Eindhoven, the Netherlands). Images were recorded using a CCD camera (MegaView II, AnalySis).

### Statistical analysis of immunogold localization

The immunogold labelling of the different anammox membranes and compartments using the antiserum directed at F-ATPase-1 was analysed statistically. Gold particles were allocated to either a membrane [outermost membrane (membrane 1), middle membrane (membrane 2) or innermost (anammoxosome) membrane (membrane 3)], when in 25 nm distance (the approximate length of the antibody-PAG-10 complex) of this membrane or to a compartment (riboplasm or anammoxosome) when in more than 25 nm distance from a membrane. Labels that were within 25 nm distance of more than one membrane (and could thus be allocated to more than one membrane) were not taken into account. Further, the paryphoplasm as a compartment was not taken into account because the distance between the outermost membrane (membrane 1) and middle membrane (membrane 2) was usually less than 50 nm, so gold particles could always be allocated to either the outermost or middle membrane of anammox bacteria. In total, 50 cells were analysed in both the incubation with the antiserum and in the incubation with the pre-immune serum. Labelling was analysed statistically using the *t*-test (GraphPad: http://www.graphpad.com/quickcalcs/) for normal distributed data with equal variances, the Welch's *t*-test for normal distributed data with unequal variances (GraphPad) or the Mann–Whitney *U*-test (VassarStats: http://faculty.vassar.edu/lowry/VassarStats.html) for non-parametric data. A *P*-value between 0.05 and 0.01 was considered significant (*), a *P*-value between 0.01 and 0.0001 very significant (**) and a *P*-value of less than 0.0001 extremely significant (***).

### Relevant accession numbers

**NCBI** (**GenBank**) (http://www.ncbi.nlm.nih.gov/)

*Kuenenia stuttgartiensis*: kuste3787 (CAJ74550) to kuste3796 (CAJ74559), kuste4592 (CAJ75354) to kuste4600 (CAJ75362), kustc0572 (CAJ71317) to kustc0579 (CAJ71324), kuste3864 (CAJ74627) to kuste3871 (CAJ74634). *Escherichia coli* K-12 MG1655: b3737 (AAC76760). *Borrelia burgdorferi* B31: bb0090 (aac66487). **Prosite**http://www.expasy.ch/prosite/): ps00605.
