# Supplementary material for: Intracellular localization of membrane-bound ATPases in the compartmentalized anammox bacterium ‘Candidatus Kuenenia stuttgartiensis’
Source: Mol Microbiol. 2010 Jun 11;77(3):701–15. doi: 10.1111/j.1365-2958.2010.07242.x (PMC2936114; doi:10.1111/j.1365-2958.2010.07242.x)
Supplement: Supplementary file 1 [file mmi0077-0701-SD1.pdf]

Intracellular localization of membrane-bound ATPases in the compartmentalized anammox bacterium "*Candidatus* Kuenenia stuttgartiensis"

Laura van Niftrik,[1*] Mary van Helden,[1] Silke Kirchen,[1] Elly G. van Donselaar,[2] Harry R. Harhangi,[1] Richard I. Webb,[3] John A. Fuerst,[3] Huub J.M. Op den Camp,[1] Mike S.M. Jetten,[1,4] and Marc Strous[1,5]

[1] Department of Microbiology, Institute for Water and Wetland Research, Faculty of Science, Radboud University Nijmegen, Heyendaalseweg 135, 6525 AJ Nijmegen, The Netherlands.

[2] Cellular Architecture & Dynamics, Utrecht University, Padualaan 8, 3584 CH Utrecht, The Netherlands.

[3] Department of Microbiology & Parasitology (JAF) / Centre for Microscopy and Microanalysis (RIW), University of Queensland, Brisbane, QLD 4072, Australia.

[4] Department of Biotechnology, Delft University of Technology, Julianalaan 67, 2628 BC Delft, The Netherlands

[5] Max Planck Institute for Marine Microbiology, Celciusstrasse 1, 28359 Bremen, Germany.

* For correspondence. E-mail L.vanNiftrik@science.ru.nl; Tel. (+31) 24 3652563; Fax (+31) 24 3652830.

Table S1. Primers designed on the catalytic beta (F-ATPases) or A (prokaryotic V-ATPase) subunit of the four *K. stuttgartiensis* ATPase gene clusters. Stop codons that were introduced in the reverse primers are indicated in bold type. ORF; open reading frame, F; forward, R; reverse, aa; amino acids.

Gene cluster	ORF	Primer	Sequence (5' → 3')	Restriction site	Fragment
F-ATPase-1	kuste3795	F	GAA TTC GTG GTA AAT ATA ACT GAA CGT AAT ATT GG	GAATTC (EcoRI)	150 aa
		R	GT CGA **CTA** ACG GGC AAA TGG GGC AAG AAG	GTCGAC (SalI)	
F-ATPase-2	kuste4592	F	GGA TCC ATG TTA CTA CAA AAA GAA ATC AAT AAA GG	GGATCC (BamHI)	147 aa
		R	GA ATT **CTA** CCT CTC AAG AGG TGC AAG CAC	GAATTC (EcoRI)	
F-ATPase-3	kustc0572	F	GGA TCC ATG GAA GGT ATC ATA GCA GCT ATT C	GGATCC (BamHI)	141 aa
		R	GA GCT **CTA** TTT GGG AAA AGG CGA AAG TAG G	GAGCTC (SacI)	
V-ATPase-4	kuste3866	F	GGA TCC ATG GGA TGT AAA TGC GGA AGC	GGATCC (BamHI)	165 aa
		R	GA GCT **CTA** AAA AGG CAC CAT GAT GCG GTG	GAGCTC (SacI)	

A. F-ATPase subunits c (gene cluster 1, 2 and 3)

```
                         10         20         30         40         50         60         70
1:kuste3790      MD-----YFV ALVIGIPVVA VAAFGCALAQ AKVVSSAVES MARQPSVAAK VQLAMIIGIA FIESLAIYSL
1:kuste3791      M-----VYFA LLAIAVSLLA IAAFGCGIGQ GIAVYGAANG MARQPDMAGK IQLVMFVGLA FIESLTIYSL
2:kuste4597      MDNVGLIGMV SIIVAGFTIA VGSIGPALGE ARAAAQALSS IAQQPDEANT ITRTLFVSMA MIESTAIYCF
3:kustc0576      MDAKTVVISV SILAAAFVMA IGGYGPAKAL GNALTEALDA TARQPEASDK IMRVLFVGMA LIESIAIYAF
E. coli          MEN--LNMDL LYMAAAVMMG LAAIGAAIGI GILGGKFLEG AARQPDLIPL LRTQFFIVMG LVDAIPMIAV
                 *                         *                  * **

                         80         90        100        110
1:kuste3790      MISFMLFGKL PKSEEVLKIF RKNTSNEELL SSAAEIVLQL SAK
1:kuste3791      MVSFILLGKL PKTEAVLEVI QHAIK----- ---------- ---
2:kuste4597      VVAMIVIFAN PFWNYVITKA GGQ------- ---------- ---
3:kustc0576      VIALIVLFAN PLIGYILK-- ---------- ---------- ---
E. coli          GLGLYVMFAV A--------- ---------- ---------- ---
```

B. Prokaryotic V-ATPase subunit L (gene cluster 4)

```
                         10         20         30         40         50         60         70
4:kuste3871      MAMDSNTVIS IGRLGAMVAL VMAAIGSCLG TGAAGAAAIG GWKKCYAQNK SAPFMLVAFV GAPLSQTIYG
B. burgdorferi   --MD------ IGLIGVNSAL TISAIGSALG MGAAGSAAIG AWKRCYMQGK PAPFLLIVFV SAPLTQIIYG
                   **       **  *   **    **** **  **** ****  ** ** * *  *** *  **  *** * ***

                         80         90        100        110        120        130        140
4:kuste3871      MILMGNIMKA AVTGAAFPVL LGAGFLGGFA MGLSAWMQGR AGAGASDALA ETGQGFGNYL MALGVIETVA
B. burgdorferi   YILMNTLYEV MMQTNPW-LL LGAGIGGGFA IAVSGFAQGK AAAGACDAFS ETGKGFATYL LVLGLIESVA
                  ***                *  ****  ****    *   **  * *** **   *** **  **   ** ** **

                        150
4:kuste3871      LFVLVFIGKT L-V
B. burgdorferi   LFVMVFLMIF KFV
                 *** **       *
```

Fig. S1. Protein alignment of the ATPase proteolipids from *K. stuttgartiensis*. **A.** Subunits c from F-ATPase-1 (kuste3790 and 3791), -2 (kuste4597) and -3 (kustc0576) aligned to the *E. coli* subunit c (b3737). **B.** Subunit L from V-ATPase-4 (kuste3871) aligned to the *B. burgdorferi* B31 subunit L (BB0090). *: conserved residues, **protonizable groups**, <u>predicted transmembrane helices</u>, PROSITE subunit c signature (PS00605; [GSTA]-R-[NQ]-P-x(10)-[LIVMFYW](2)-x(3)-[LIVMFYW]-x-[DE]), inconsistencies in signature motifs.

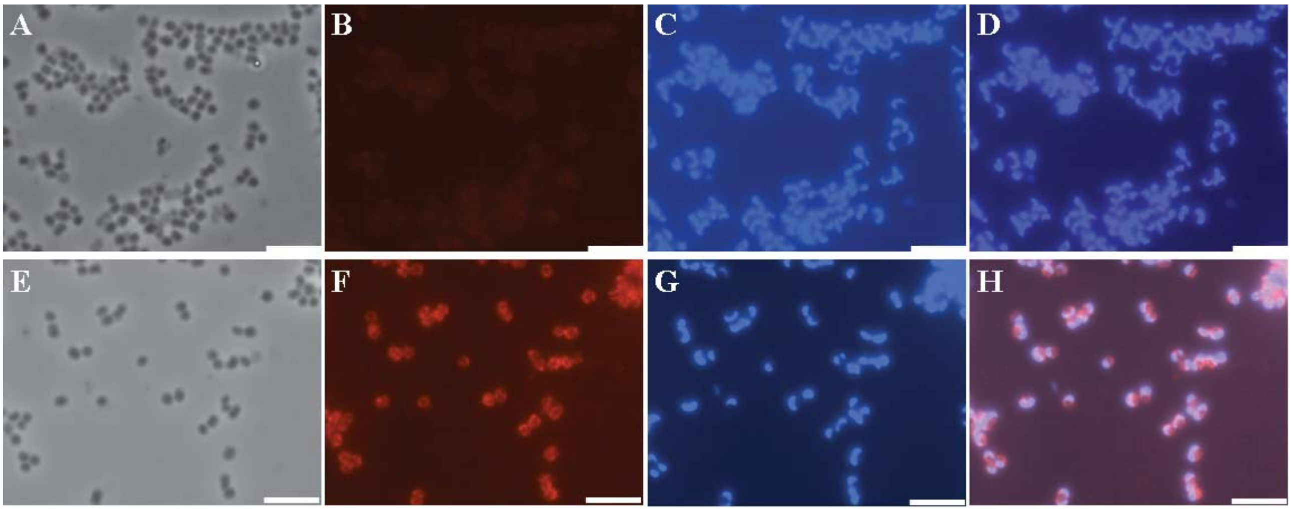

Fig. S2. Immunofluorescence analysis of formaldehyde-fixed *K. stuttgartiensis* cells using the pre-immune (A-D) and antiserum (E-H) directed at the catalytic beta subunit of the F-ATPase-1 gene cluster found in the *K. stuttgartiensis* genome. A&E; phase contrast, B&F; Cy3, C&G; DAPI, D&H; Cy3+DAPI, Cy3 exposure time; 600 ms, Scale bars; 5 µm.

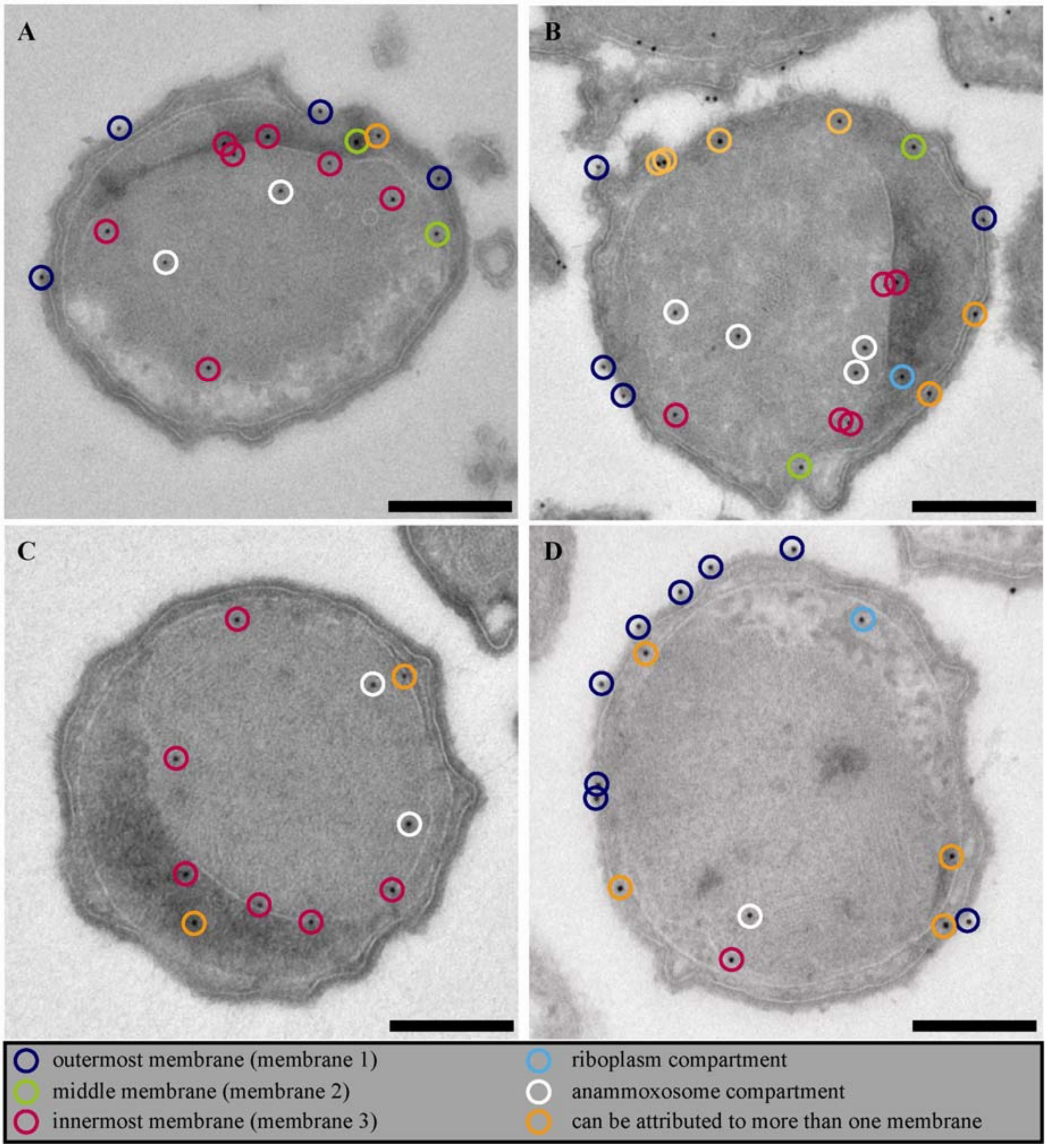

Fig. S3. Annotation of specific gold particles in the immunogold localization of *K. stuttgartiensis* rehydrated cryosections using the antiserum directed at the catalytic beta subunit of the F-ATPase-1 gene cluster. Gold particles were allocated to either a membrane (outermost membrane 1, middle membrane 2 or innermost membrane 3) when in 25 nm distance (the approximate length of the antibody-PAG-10 complex) of this membrane or to a compartment (riboplasm or anammoxosome) when in more than 25 nm distance from a membrane. Labels that were within 25 nm distance of more than one membrane (and could thus be allocated to more than one membrane) were not taken into account. Further, the paryphoplasm as a compartment was not taken into account because the distance between the outermost membrane (membrane 1) and middle membrane (membrane 2) was usually less than 50 nm, so gold particles could always be allocated to either the outermost or middle membrane of the anammox cell.
